# Two-photon dual imaging platform for *in vivo* monitoring cellular oxidative stress in liver injury

**DOI:** 10.1038/srep45374

**Published:** 2017-03-28

**Authors:** Haolu Wang, Run Zhang, Kim R. Bridle, Aparna Jayachandran, James A. Thomas, Wenzhu Zhang, Jingli Yuan, Zhi Ping Xu, Darrell H. G. Crawford, Xiaowen Liang, Xin Liu, Michael S. Roberts

**Affiliations:** 1 Therapeutics Research Centre, School of Medicine, The University of Queensland, Princess Alexandra Hospital, Woolloongabba, QLD 4102, Australia; 2 Department of Biliary-Pancreatic Surgery, Ren Ji Hospital, School of Medicine, Shanghai Jiao Tong University, 1630 S. Dongfang Road, Shanghai, 200127, China; 3 Australian Institute for Bioengineering and Nanotechnology, The University of Queensland, St Lucia, QLD 4072, Australia; 4 School of Medicine, The University of Queensland, Gallipoli Medical Research Institute, Greenslopes Private Hospital, Greenslopes, QLD 4120, Australia; 5 Department of Gastroenterology, The Prince Charles Hospital, School of Medicine, The University of Queensland, Chermside, QLD 4032, Australia; 6 State Key Laboratory of Fine Chemicals, School of Chemistry, Dalian University of Technology, Dalian, 116024, China; 7 School of Pharmacy and Medical Science, University of South Australia, Adelaide, SA 5001, Australia

## Abstract

Oxidative stress reflects an imbalance between reactive oxygen species (ROS) and antioxidants, which has been reported as an early unifying event in the development and progression of various diseases and as a direct and mechanistic indicator of treatment response. However, highly reactive and short-lived nature of ROS and antioxidant limited conventional detection agents, which are influenced by many interfering factors. Here, we present a two-photon sensing platform for *in vivo* dual imaging of oxidative stress at the single cell-level resolution. This sensing platform consists of three probes, which combine the turn-on fluorescent transition-metal complex with different specific responsive groups for glutathione (GSH), hydrogen peroxide (H_2_O_2_) and hypochlorous acid (HOCl). By combining fluorescence intensity imaging and fluorescence lifetime imaging, these probes totally remove any possibility of crosstalk from *in vivo* environmental or instrumental factors, and enable accurate localization and measurement of the changes in ROS and GSH within the liver. This precedes changes in conventional biochemical and histological assessments in two distinct experimental murine models of liver injury. The ability to monitor real-time cellular oxidative stress with dual-modality imaging has significant implications for high-accurate, spatially configured and quantitative assessment of metabolic status and drug response.

Oxidative stress has been reported as an early unifying event in the development and progression of various diseases including injury^1^,^2^, cancer^3^, and many inflammatory diseases^4^. It reflects an imbalance between the production of reactive oxygen species (ROS) and antioxidant defenses, such as glutathione (GSH)^5^. Excessive production of ROS damages all components of the cell, including lipids, proteins, and DNA. Some ROS, including hydrogen peroxide (H_2_O_2_) and hypochlorous acid (HOCl), also act as cellular messengers, and can cause disruptions in normal mechanisms of cellular signaling^5^. While GSH, the major ROS-scavenging system in cells to reduce ROS stress, can detoxify the reactive intermediates and repair the resulting damage. Because ROS and antioxidants have distinct sources of production and are particularly sensitive to upstream molecular interventions^6^, their detection at the single cell-level resolution could be useful for identifying subpopulations of cells with different susceptibility to ROS-induced injury in different stages of diseases. Moreover, the oxidative stress endpoints can report early and molecular changes due to treatment^7^, and have potential to serve as powerful biomarkers of drug response. For example, in liver diseases, the primary endpoint of drug efficacy is functional recovery of hepatocytes. The current treatment evaluations include imaging liver morphology, monitoring blood levels of liver enzymes, bilirubin and markers of inflammation, and assessing the signs and symptoms^8^. Yet each of these current techniques fails to capture dynamic changes in metabolic state and poorly reflects sensitivity to drug efficacy. Cellular and molecular changes of hepatocytes precede changes in liver morphology or markers in peripheral blood during treatment. If these molecular endpoints can be identified and measured, they would provide powerful biomarkers for early-drug response.

Methods to detect oxidative stress *in vivo* have encountered technical challenges, which prevented implementation of this method for preclinical drug efficacy screening^2^,^9^. A number of GSH and ROS-detection probes have been developed^9^,^10^,^11^,^12^,^13^. Most of them are single-mode intensity-based probes, which can provide quantitative results, but may often be influenced by fluid optical properties, endogenous fluorophores, probe concentration, and other *in vivo* environmental or instrumental factors. The fluorescence lifetime of probes are independent of these interfering factors, offering accurate and ultrasensitive detecting the presence of many components of cell signaling pathways^14^,^15^,^16^. Thus, the combination of fluorescence intensity imaging and fluorescence lifetime imaging (FLIM) is an ideal procedure for intracellular oxidative stress investigations with high reliability and accuracy. Up to now, however, no such dual-mode probe has been developed for *in vivo* real-time molecular imaging. We have previously synthesized a transition-metal complex-based sensing platform for detecting cellular GSH and ROS levels *in vitro*^17^,^18^,^19^,^20^. This sensing platform consists of three probes, which exhibit favorable photophysical properties including high photostability, selective and quantitative response. To further investigate the fluorescence lifetime and two-photon absorption behavior of this platform, and advance to monitoring multiple analytes at the single cell-level resolution in living systems, here we combined fluorescence intensity imaging and FLIM to detect oxidative stress in the liver of living mice using this sensing platform. We investigated this approach as a tool for monitoring cellular oxidative stress to predict drug response in two of the most common types of liver injury: acetaminophen (APAP)-induced liver injury, which accounts for approximately one-half of all acute liver failures in the western world today^21^, and hepatic ischemia-reperfusion (I/R) injury, which often occurs during liver resection and liver transplantation^22^. The drug response predicted by cellular oxidative stress was further compared with alanine aminotransferase (ALT) levels in blood and histological examination of the liver. This work represents a significant advancement in the tools available to study cellular metabolism and predict drug response in living systems.

## Results

### Sensing mechanism of the two-photon dual imaging probes

**Figure 1:**
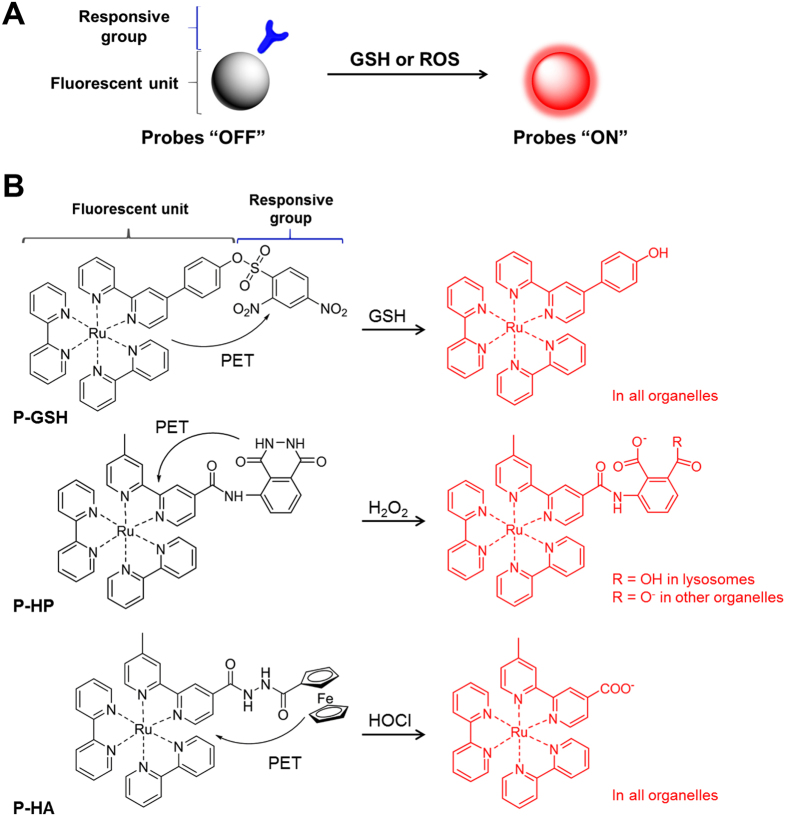
Design of two-photon sensing platform for imaging of oxidative stress. (**A**) Illustration of the sensing mechanism. This sensing platform consists of two parts: a ruthenium complex as the turn-on fluorescent unit, and a responsive group as the GSH, H_2_O_2_ or HOCl reaction moiety. (**B**) Response reaction of P-GSH, P-HP and P-HA toward GSH, H_2_O_2_ or HOCl, respectively. The probes are non-fluorescent due to the effective photo-induced electron transfer process (PET). In the presence of GSH, H_2_O_2_ or HOCl, the responsive group can be quantitatively cleaved, and the reaction ruthenium complex will become highly fluorescent. The protonation state of P-HP depends on the organelle pH values. The counter-ions are sodium and potassium in biological conditions.

This two-photon sensing platform consists of three probes (Fig. 1A), which combine a tris(2,2′-bipyridine)Ru(II) complex as the turn-on fluorescent unit, with the specific responsive group for GSH, H_2_O_2_ or HOCl, which also serves as an electron acceptor. Excitation of the tris(2,2′-bipyridine)Ru(II) complex leads to the triplet state of metal-to-ligand charge transfer (^3^MLCT), and by this process, the metal electrons are transferred to the bipyridine ligands in an emissive state. As shown in Fig. 1B, when this ligand is conjugated to a strong electron acceptor, such as phenyl-2,4dinitrobenzenesulfonate (phenyl-DNBSO) in the GSH-detection probe (P-GSH), the electron transfer destination will be diverted from 2,2′-bipyridine to phenyl-DNBSO. Thus, the ^3^MLCT is corrupted and the fluorescence and lifetime of the Ru(II) complex are quenched by an intramolecular photo-induced electron transfer (PET) process. While the reaction with GSH, H_2_O_2_ or HOCl can specifically trigger the quantitative cleavage of the electron acceptor group, and the PET process is eliminated, then the fluorescence of the ruthenium complex can be turned on.

### *In vitro* characterization of the two-photon dual imaging probes

**Figure 2:**
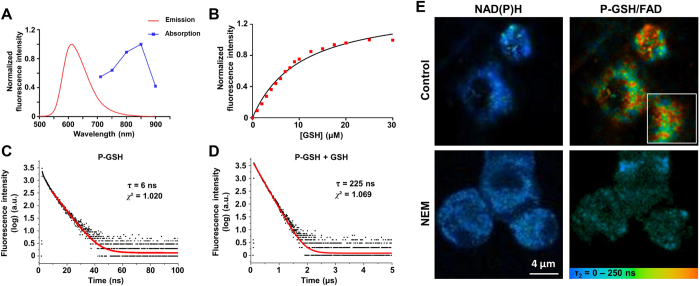
Spectral characterization of P-GSH *in vitro*. (**A**) Two-photon absorption and emission spectra of P-GSH reacted with 10 μM of GSH in PBS buffer. (**B**) Fluorescence response of P-GSH (10 μM) to varying concentrations of GSH. (**C**) Emission decay of P-GSH in PBS buffer. (**D**) Emission decay of P-GSH in PBS buffer with GSH (20 μM). (**E**) FLIM images of representative P-GSH loaded AML12 cells with (lower row) or without (upper row) NEM treatment. The autofluorescence of NAD(P)H was collected at λ_Exc_/λ_Em_: 740/350 to 450 nm. The fluorescence signal for probes and FAD was collected at λ_Exc_/λ_Em_: 850/515 to 620 nm. Values are the mean for *n* = 5 replicates.

We have previously reported that the ruthenium complex has a broad single-photon absorption spectrum from 350 to 550 nm^17^,^18^,^19^,^20^. For *in vivo* application of deep-tissue imaging, we first evaluated the two-photon absorption spectrum of these two-photon dual imaging probes. Figure 2A shows the two-photon absorption spectrum of P-GSH with a peak at 850 nm, and its emission spectrum with a peak at 612 nm. To determine the fluorescence intensity of P-GSH in response to GSH, we added GSH in a stepwise manner and measured the fluorescence signal in an emission channel of 515–620 nm at a two-photon excitation wavelength of 850 nm. The dose-dependent intensity enhancement of P-GSH showed good linear relationships in the concentration range from 0 to 10 μM of GSH, and the maximum intensity was at the concentration of 20 μM (Fig. 2B). The H_2_O_2_ -detection probe (P-HP) and HOCl-detection probe (P-HA) have similar excitation and emission spectra to that of P-GSH (Supplementary Fig. 1A and B). Good linear correlations can be obtained in the concentration range from 0 to 50 μM of H_2_O_2_ and 0 to 40 μM of HOCl (Supplementary Fig. 1C and D). Because of the differences in specific responsive groups and PET process, the P-GSH, P-HP and P-HA have different sensitivity for detection of GSH, H_2_O_2_ and HOCl. Metal complex-based probes have been reported to be particularly suitable for FLIM imaging because their fluorescence lifetime (typically more than 50 ns) is much longer than those of tissue (2–3 ns) and most organic dyes (1–5 ns)^23^. So we further measured the fluorescence lifetime of P-GSH in PBS. The P-GSH has a characteristic 38-fold increase of emission lifetime from 6 to 225 ns in the presence of GSH (Fig. 2C and D). P-HP and P-HA also have over 20-fold increase of emission lifetime from 5.5 and 4.3 ns to 146 and 90.5 ns, respectively. The optical characteristics of P-GSH, P-HP and P-HA are summarized in Supplementary Table. 1.

We have previously reported the specificity and *in vitro* cellular uptake properties of these probes^17^,^18^,^19^,^20^. To further examine their applicability for detecting cellular GSH and ROS levels using FLIM, probes (30 μM) were incubated with a mouse hepatocyte cell line (AML12) in culture media for 2 h. Figure 2E displays the spatial distribution of the fluorescence lifetime signal of P-GSH in the AML12 cells in two emission channels. According to our previous work, long-lifetime fluorescent probes can be differentiated *in vivo* using the slow decay lifetime τ_2_, rather than the fast decay lifetime τ_1_^24^. Thus, in this study, the pseudo-color was based on the slow decay lifetime (τ_2_) of individual pixel. In the 350–450 nm spectral channel (left), the fluorescence signal mainly comes from nicotinamide adenine dinucleotide phosphate (NAD(P)H), which is a major endogenous fluorophore in cells. The spectral channel of 515–620 nm (right) captured the fluorescence signal of P-GSH, as well as aufluorescence signal from flavin adenine dinucleotide (FAD) in cells. Characteristic longer slow decay lifetimes (>100 ns) could be detected within the cells after P-GSH administration in the 515–620 nm spectral channel. While no such long lifetime was observed after cells incubated with a thiol scavenger, N-ethylmaleimide (NEM), to remove the endogenous GSH. Because reacted probe has much longer lifetime compared to unreacted probe and FAD, we believe the significant increase of τ_2_ with the cells after probe administration is due to the reaction of probe and their specific substrate. These results confirm that the cellular lifetime change is attributed to the reaction of P-GSH with endogenous intracellular GSH.

To investigate the photostability of P-GSH, P-HP and P-HA for *in vivo* imaging, we next incubated these probes in PBS, GSH, H_2_O_2_ or HOCl under irradiation with a deuterium lamp at room temperature, whilst fluorescence intensity and fluorescence lifetime was assessed over time. There was no significant change in the fluorescence intensity (Supplementary Fig. 2) or fluorescence lifetime during the 2 hour irradiation. Thus, our dual imaging probes exhibit characteristic long emission lifetime with two-photon excitation and high photostability required for detecting GSH or ROS levels in pathophysiological conditions *in vivo*.

### Imaging of cellular oxidative stress in APAP-induced liver injury

The mechanism of APAP-induced liver injury is well established. An overdose of APAP can lead to oxidative stress through the overproduction of ROS (mainly H_2_O_2_). This results in the consumption of key cellular antioxidants, such as GSH, initiating a signaling cascade that results in necrotic cell death. GSH and the GSH precursor N-acetylcysteine (NAC) can scavenge the reactive metabolites, and have been used to treat patients with APAP overdose^25^,^26^. Thus APAP-induced liver injury serves as an ideal model of investigating the potential of cellular oxidative stress for early prediction of treatment response in a clinically relevant model.

**Figure 3:**
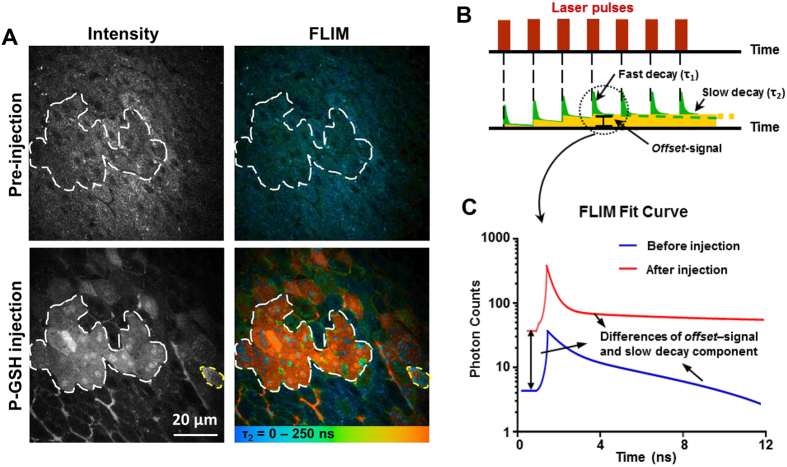
Dual-mode *in vivo* imaging of GSH in hepatocytes of mice after APAP administration. (**A**) Representative fluorescence intensity and FLIM images of mouse liver before and 15 min after probes injection. All images were recorded at λ_Exc_/λ_Em_: 850/515 to 620 nm. Scale bar: 20 μm. (**B**) Sketch to illustrate the parameters of the fit procedure of the fluorescence decay curve. Two lifetimes, τ_1_ and τ_2_ represent the fast and slow decay lifetimes. The slow decaying fluorescence accumulates on repeated laser pulsing to create enhanced background signal, defined as ‘*offset*’. (**C**) Fluorescence decay fit curves of representative area (circled in white) before (Blue) and after (Red) P-GSH injection.

We anesthetized the mice and exposed the liver at 60 min after APAP administration. Fluorescence intensity imaging and FLIM were acquired at 15 min after injection of 50 μM of P-GSH or P-HP into the portal vein (Supplementary Fig. 3). As shown in Fig. 3A, signals induced by the probes response to GSH in cells were clearly observed using both fluorescence intensity imaging and FLIM methods. A significant increase in P-GSH fluorescence was observed in some hepatocytes (circled in white). Further analysis of FLIM data reveals a significant increase (>100 ns) in the slow decay lifetime of this area after injection of P-GSH, which is responsible for the change of lifetime after reacting with GSH (Fig. 3B and C). Some false-positive hepatocytes determined by fluorescence intensity imaging (circled in yellow in Fig. 3A) were found with shorter fluorescence lifetime using FLIM, which removes all the possibilities of crosstalk from *in vivo* environmental or instrumental factors. There was no significant decrease of probe signals for up to 2 hours after injection. We then compared P-GSH to bromobimane, a fluorescent heterocyclic compound commonly used for GSH sensing^27^,^28^. The fluorescence signal produced by bromobimane cannot be differentiated from the liver autofluorescence *in vivo* after intraportal injection. Comparable fluorescence intensity imaging of GSH can only be obtained from liver sections treated with bromobimane (Supplementary Fig. 4). The percentage of GSH-positive hepatocytes has no significant difference between this detection method and FLIM (Supplementary Table 2, *p* > 0.05). Thus these *ex vivo* imaging results using bromobimane confirmed the *in vivo* results using P-GSH. While the photochemical characteristics of bromobimane based imaging restrict its utility to *ex vivo* tissue in contrast to *in vivo* P-GSH imaging of the GSH generation in the liver injury.

**Figure 4:**
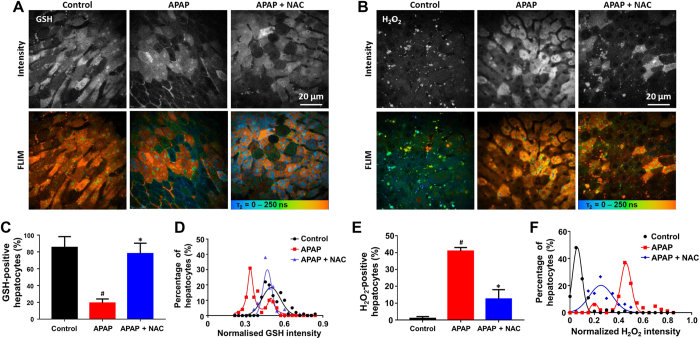
Dual-mode quantitative imaging of the change of oxidative stress in hepatocytes responses to NAC treatment against APAP induced liver injury. (**A**,**B**) Representative fluorescence intensity and FLIM images of cellular GSH or H_2_O_2_ of the control, APAP, APAP + NAC groups at 30 min after NAC treatment (75 min after APAP administration). All images were recorded at λ_Exc_/λ_Em_: 850/515 to 620 nm. Scale bar: 20 μm. (**C**,**E**) The percentages of GSH or H_2_O_2_-positive hepatocytes of the control, APAP, APAP + NAC groups. (**D**,**F**) Population density modeling of the mean GSH or H_2_O_2_ intensity per cell in control APAP, APAP + NAC groups. Values are the mean ± s.d. for *n* = 5 mice; *p < 0.05, compared with APAP groups; ^#^*p* < 0.05, compared with control group.

Having quantified the cellular oxidative stress response following APAP-induced liver injury, we next evaluated the therapeutic context. As shown in Supplementary Fig. 5, zonal GSH changes in the liver during NAC treatment were identified in fluorescence intensity images and FLIM at low magnification. Representative dual-mode images of GSH and H_2_O_2_ at high magnification (Fig. 4A and B) demonstrate the high-resolution capability of this technique, which allows single-cell analysis (Supplementary Fig. 6) and population modeling for quantification of cellular subpopulations with varying oxidative stress. Calculated from FLIM images, we found that NAC treatment significantly increased the percentage of GSH-positive hepatocytes, and decreased that of H_2_O_2_-positive hepatocytes, compared with the untreated (*p* < 0.05, Fig. 4C and E). Population density modeling of cellular distributions of the GSH intensity calculated from fluorescence intensity images revealed two populations of GSH-positive hepatocytes at 75 min after APAP administration, both lower than the mean intensity of the controls (Fig. 4D). The NAC treated group has a single population with higher fluorescence intensity maxima at 30 min after NAC administration. This heterogeneity of cellular GSH level was not seen after NAC treatment, suggesting that the APAP sensitive cell subpopulation responses to NAC treatment. The comparison of H_2_O_2_ intensity determined on a per cell basis showed similar distributions between groups as to GSH intensity (Fig. 4F).

**Figure 5:**
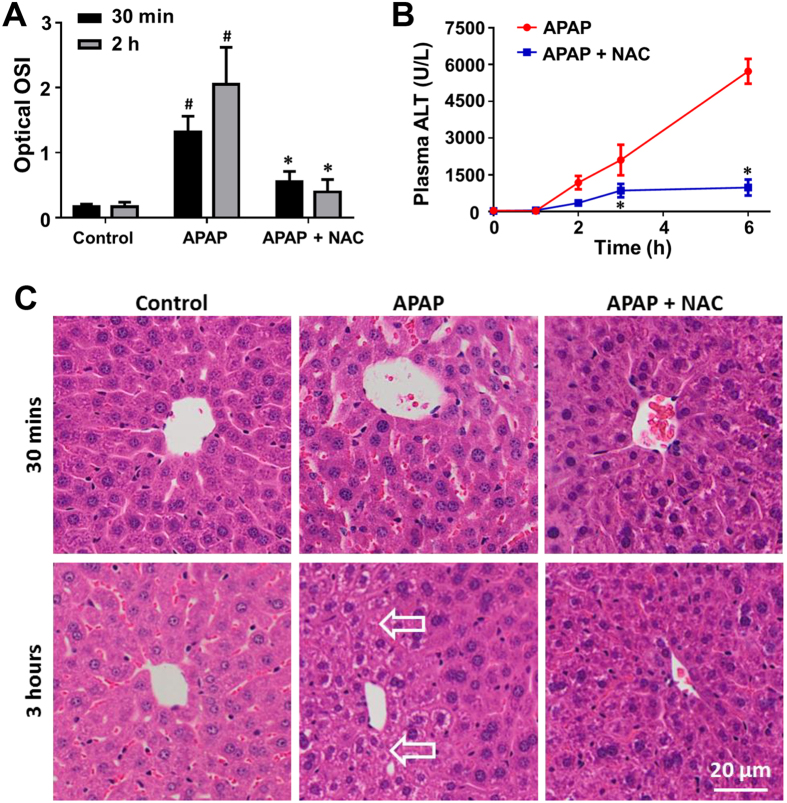
Optical oxidative stress index (OSI) of the liver detects response to NAC treatment against APAP induced liver injury. (**A**) Optical OSI of the liver at 30 min and 2 hours after NAC treatment (75 and 165 min after APAP administration). (**B**) Concentration-time profile of ALT levels in peripheral blood of the APAP and APAP + NAC groups. (**C**) Representative histology (H&E staining) of the liver of the control, APAP, APAP + NAC groups at 30 min and 3 hours after NAC treatment (75 and 225 min after APAP administration). Arrows indicate cellular necrosis. Values are the mean ± s.d. for *n* = 5 mice; *p < 0.05, compared with untreated groups; ^#^*p* < 0.05, compared with control group.

A composite endpoint, the optical oxidative stress index (OSI), was computed as the ratio of the mean intensity of cellular ROS to the mean intensity of cellular GSH^29^. After 30 min of NAC treatment, the optical OSI was significantly reduced compared with the untreated (*p* < 0.05, Fig. 5A). By 2 hours, the optical OSI further decreased in the treatment group (*p* < 0.05). The imaging results were compared with conventional biochemical and histological assessment. As shown in Fig. 5B, a significant reduction in plasma alanine aminotransferase (ALT) levels associated with NAC treatment was first detected 3 hours after therapy. Furthermore, an increase beyond control levels was not detected until 2 hour after delivery of APAP. Histological changes were not apparent 30 min after NAC treatment (Fig. 5C). Remediation of APAP-induced hepatocellular necrosis was observed in hematoxylin and eosin (H&E) stained sections of liver tissue 3 hours after NAC treatment. The optical OSI changes therefore precede the conventional measures such as plasma liver enzyme levels or histological features of liver injury. In addition, no obvious necrosis and abnormity were observed in the sections of liver, kidney and spleen tissue from the control group (Supplementary Fig. 7), suggesting that the two-photon dual imaging probes have no distinct toxicity. Altogether, these results confirm the utility of this sensing platform for monitoring cellular oxidative stress to predict drug response to APAP-induced liver injury *in vivo*.

### Imaging of cellular oxidative stress in hepatic ischemia-reperfusion injury

**Figure 6:**
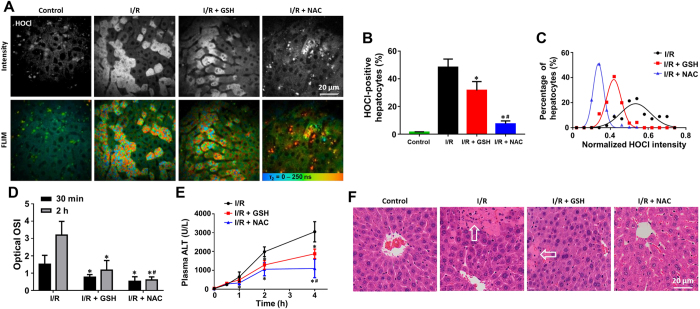
Quantitative *in vivo* detection of different responses to GSH and NAC treatment against hepatic I/R injury. (**A**) Representative fluorescence intensity and FLIM images of cellular HOCl of the control, I/R, I/R + GSH, and I/R + NAC groups at 30 min after reperfusion. All images were recorded at λ_Exc_/λ_Em_: 850/515 to 620 nm. Scale bar: 20 μm. (**B**) The percentages of HOCl-positive hepatocytes in all groups. (**C**) Population density modeling of the mean HOCl intensity per cell in the I/R, I/R + GSH, and I/R + NAC groups. (**D**) Optical OSI of the liver at 30 min and 2 hours after reperfusion. (**E**) Concentration-time profile of ALT levels in peripheral blood of the I/R, I/R + GSH, and I/R + NAC groups. (**F**) Representative histology (H&E staining) of the liver of all groups at 4 hours after reperfusion. Arrows indicate cellular necrosis. Values are the mean ± s.d. for *n* = 5 mice; *p < 0.05, compared with untreated groups; ^#^*p* < 0.05, compared with I/R + GSH group.

It is well documented that during hepatic I/R injury, ROS (mainly HOCl) are generated by neutrophils and diffuse into hepatocytes, causing oxidant stress-mediated injury^30^. This pathway can be inhibited by administration of GSH or NAC^31^. Therefore, we used a mouse hepatic I/R injury as an independent model to test the potential of evaluating cellular oxidative stress for early prediction of treatment response. At 30 min after reperfusion, we observed reductions in the percentage of HOCl-positive hepatocytes and mean HOCl intensity in both GSH and NAC treated groups using P-HA (Fig. 6A–C), indicating successful remediation of cellular ROS. There were differences in treatment response between GSH and NAC. Consistent with reports that GSH is inferior to NAC as an antidote to hepatic I/R injury^31^, GSH treatment resulted in production of higher HOCl level compared to the NAC treated group though still lower than the untreated group (*p* < 0.05, Fig. 6B and C). Additionally, GSH and NAC increased cellular GSH to the same extent (Supplementary Fig. 8).

The optical OSI significantly decreased in both GSH and NAC treated groups compared with the untreated group from 30 min after reperfusion (*p* < 0.05, Fig. 6D). The difference in optical OSI between GSH and NAC treated groups was observed at 2 hours after reperfusion (*p* < 0.05). However, no difference in ALT levels in peripheral blood was detected between the treatment groups within 4 hours after reperfusion (Fig. 6E). As shown in Fig. 6F, the drug response predicted by dual-mode quantitative imaging was consistent with reduced hepatocyte necrosis observed in H&E stained sections of liver tissue. The optical OSI changes therefore precede the conventional measures such as plasma liver enzyme levels or histological features of hepatic I/R injury. These results fully characterize optical OSI and show its potential for monitoring early-drug response *in vivo* at the single-cell level.

## Discussion

Oxidative stress contributes to a diverse array of physiological and pathological events in living organisms, but there is an insufficient understanding of how its cellular fluxes initiate signaling cascades in living animals in stages of health, aging, and disease^32^. Whereas a growing number of chemical tools have been developed to probe redox biology, technologies that can monitor fluctuations in cellular redox environment in living animals remain limited. We showed that our transition-metal complex-based sensing platform is capable of simultaneous two-photon fluorescence intensity imaging and FLIM, enabling accurate *in vivo* detection of ROS and GSH at the single cell-level resolution. Moreover, the imaging results correlate well with conventional markers of liver injury such as liver enzyme levels and histology, which validate the optical OSI as a prodromal imaging biomarker for prediction and evaluation of drug efficacy.

Although molecular probes have been widely used in biomedical imaging, most of these studies exclusively involve steady-state emission where changes in intensity or emission energy of the probes are used as the imaging signal^23^. Metal complex-based probes are particularly suitable for lifetime-based imaging in a wide range of targets in cells, as they emit from long-lived, triplet-based excited states that are usually efficiently populated through the heavy-atom effect. For example, some metal complex-based probes can cross the membrane and accumulate in the mitochondria to detect metabolic status in mitochondria of cells^33^. Ruthenium-based probes have been developed for lifetime-based imaging of the cellular DNA, RNA and oxygen levels^23^,^34^. Furthermore, metal complexes often possess high two-photon absorption cross-sections, making them particularly compatible with two-photon based lifetime microscopy techniques^35^. In particular, the π-conjugated ligands of our metal complex-based probes endow them with two-photon absorption property for *in vivo* deep-tissue imaging. Moreover, the rapid sensing kinetics and large dynamic range of sensitivity allow our metal complex-based probes to detect nanomolar to micromolar levels of ROS and GSH in near–real time. This permits sensitive monitoring of cellular oxidative stress levels as a mediator of liver injury. This sensing platform has advantages over small-molecule fluorescent-based probes for *in vivo* ROS or GSH imaging reported to date. Conventional emission-based detection methods are influenced by probe concentration and the biomolecular microenvironment^10^,^36^,^37^, or have only been employed under one-photon model^9^,^38^. We found metal complex-based probes can uniquely overcome current limitations to improve detection accuracy in quantitative visualization of oxidative stress by FLIM. In addition, since these probes do not have significant systemic toxicity in animals, they may also be applied to study the etiology and pathophysiology of diseases involving oxidative stress. It is also worth mentioning that the chief limitation of these probes is their irreversibility. Once all reacted, they will not report any more changes of the actual GSH, H_2_O_2_ and HOCl concentrations. Therefore, reversible probes such as conjugated polymer based phosphorescent nanoparticles will be highly needed in the future for *in vivo* quantitative measurement^39^. Another limitation of this study is the short measurement time window of FLIM (12.5 ns) in our multiphoton microscopy (LaVision and DermaInspect). Although we do have detected significant increases in the slow decay lifetime within the cells after probe administration, phosphorescence lifetime detection is a much better suitable technique to accurately determine the long fluorescence lifetime of the probes.

Current methods of evaluation of drug response are generally based on organ morphology, histological characteristics, and biomarkers in peripheral blood. Molecular changes induced by treatment precede changes in morphology or these biomarkers, and may provide proximal endpoints of drug response^40^. The optical OSI from dual-mode images using metal complex-based probes captures these drug-induced changes in GSH and ROS, which have been hypothesized to correlate closely with treatment outcomes^6^,^41^. Our study introduce the concept of molecular imaging as a tool to study drug response *in vivo*, and is the first to correlate optical OSI with a standard assay of drug response in liver injury. The data presented here support the use of optical OSI as an effective imaging biomarker for evaluation of drug response, as cellular H_2_O_2_, HOCl and GSH levels change at an early stage after liver injury, and precede even histological signs of liver tissue and changes of liver enzymes in peripheral blood. Beyond the study of liver injury, optical OSI may also have utility predicting drug response in other systems. For example, in neurodegenerative diseases, ROS have been implicated as the initiators of protein misfolding and the downstream inducers of cell death^42^. ROS also have an important role in inflammatory diseases, acting as effectors and signaling molecules in both the innate and adaptive immune response^7^. ROS levels change rapidly during anti-cancer therapy. High levels of ROS generated by chemotherapeutic agents for liver cancer, such as doxorubicin, platinum drugs and 5-fluorouracil, can induce cancer cell death^43^,^44^. Thus, detection of oxidative stress in real time and with high resolution using metal complex-based probes may help uncover mechanisms of ROS production and action in a broad range of diseases and contribute to the development of new therapeutics.

High-resolution image analysis revealed initial heterogeneous GSH and ROS levels among hepatocytes after APAP-induced injury (Fig. 4D and F). The cellular details of the liver can be imaged deep to 250 μm below the fibrous capsule of Glisson using multiphoton microscopy. According to our previous study, imaging depth of 50 to 100 μm was the clearest for observing cellular and subcellular morphology in the liver^24^,^45^. Hepatocytes around the central vein (Zone 3) are more sensitive to APAP-induced injury than those around the portal vein (Zone 1)^46^. Consistent with this, our data suggest that there exist two intrinsic subpopulations of hepatocytes with differential tolerance to APAP toxicity. This heterogeneity of cellular GSH and ROS levels was not seen after NAC treatment, suggesting that NAC is protective to this APAP sensitive subpopulation. The ability to detect disease severity at a cellular level before treatment may provide leads for identification of drugs that target such susceptible subpopulations before they are selected by the primary therapy.

In conclusion, in this proof-of-concept study using a two-photon sensing platform, we present the first dual-mode quantitative imaging of cellular oxidative stress *in vivo*. The high resolution and high accuracy of imaging allows subpopulation analysis for identification of heterogeneous disease severity among cell populations and assessment of drug response. We demonstrate that optical OSI can be used as an early and sensitive indicator of metabolic response to treatment in two distinct models of liver injury. Altogether, these results suggest that two-photon dual imaging probes are a powerful tool to monitor the production and action of cellular oxidative stress in a broad range of diseases and inform the rational modification of treatment decisions accordingly.

## Methods

### Chemicals and cells

All chemicals were obtained from Sigma-Aldrich (St Louis, MO, USA) unless otherwise stated. Bromobimane was purchased from Santa Cruz (Santa Cruz, California, USA). PBS was purchased from Invitrogen (Carlsbad, CA, USA). The P-GSH, P-HP and P-HA were synthesized according to the literature^17^,^18^,^19^,^20^. AML12 cells were obtained from ATCC (Manassas, VA, USA) and maintained *in vitro* under cell culture conditions recommended by ATCC.

### *In vitro* characterisation

Two-photon absorption spectrum was recorded using DermaInspect system (Jen-Lab GmbH, Jena, Germany). The power (15 mW) and laser pulses (80-MHz and 85 fs pulse width) were adjusted under the chosen measurement conditions that were kept constant throughout this study. Emission spectra were measured on a Perkin-Elmer LS 50B fluorescence spectrometer with excitation and emission slits of 10 nm. To determine optical responses of probes toward different GSH, H_2_O_2_ and HOCl in solution, the fluorescence intensities (λex = 850 nm, λem = 515 to 620 nm) of the P-GSH, P-HP and P-HA (10 μM) in PBS (30 mM, pH = 7.4) were measured 5 min after the addition of GSH, H_2_O_2_ and HOCl to determine the intensity enhancement. Emission lifetimes were measured on an ISS-Chronos multifrequency cross-correlation phase and modulation lifetime spectrometer (ISS Inc., Champaign, IL, USA). For examining the applicability of probes for detecting cellular GSH and ROS levels using FLIM imaging, 1 × 10^5^ AML12 cells per well were plated in 12 well plates and incubated for 24 h at 37 °C and 5% CO2. To remove the intracellular GSH, cells were incubated in serum free medium containing N-ethylmaleimide (100 μM) for 1 h at 37 °C in the incubator. Cells were then exposed to 30 mM of P-GSH in serum free medium and incubated for 2 h in the same conditions. To investigate the photostability of P-GSH, P-HP and P-HA, these probes were incubated in PBS, GSH, H_2_O_2_ or HOCl under irradiation with a 30 W deuterium lamp (one-photon spectrum: 85 to 400 nm) at room temperature. Fluorescence intensity and FLIM imaging was performed using DermaInspect system (Jen-Lab GmbH, Jena, Germany) equipped with an ultrashort (85 fs pulse width) pulsed mode-locked 80-MHz titanium sapphire laser (MaiTai, Spectra Physics, Mount View, CA, USA) and a time-correlated single-photon counting (TCSPC) SPC-830 detector (Becker & Hickl, Berlin, Germany). The excitation wavelength was set to 740 nm for autofluorescence and 850 nm for probe signals, with emission signal ranges of 350 to 450 nm and 515 to 620 nm established respectively through the use of BG39 bandpass filters (BG39, Schott glass color filter, Schott MG, Mainz, Germany). Images were recorded with oil-immersion × 40 objectives (Carl Zeiss, Oberkochen, Germany). The laser power was set to 15 mW for × 40 magnification imaging, and the acquisition time for obtaining the images was 7.4 seconds per frame. Fluorescence emission was spectrally resolved between linearly arranged photon counters through the use of dichroic filters in the beam path.

### Animal models

Male 8-week-old BALB/c mice were purchased from the Animal Resource Centre (Perth, Western Australia). All animal procedures were approved by the Animal Ethics Committee of the University of Queensland and were carried out in accordance with Australian Code for the Care and Use of Animals for Scientific Purposes 8th edition. For APAP-induced liver injury, mice received gavage of 500 mg/kg APAP in 0.9% saline. Liver ischemia-reperfusion injury was induced by clamping the portal vein and hepatic artery supplying the median and left lobes using a microvascular clamp. After 45 min of partial ischemia, the clamp was removed to allow reperfusion in the liver. For drug efficacy studies, animals were treated with 150 mg/kg of NAC intravenously 45 min after APAP administration^2^, and 200 mg/kg of GSH or 150 mg/kg of NAC intravenously 45 min before liver ischemia^47^.

### *In vivo* imaging of GSH and ROS

Mice were anaesthetized initially by the intraperitoneal injection of ketamine hydrochloride (80 mg/kg) and xylazine (10 mg/kg). Body temperature was controlled by placing mice on a heating pad set to 37 °C. Intravital imaging of the mouse liver was performed as previously described^24^,^45^. Briefly, a midline laparotomy is performed and the liver is exposed for imaging. The left lobe of the liver is placed on the metal plate, which was slightly raised above the intraperitoneal cavity to minimize pressure on the organs underneath. Normal saline was used to keep the liver moist and attached to the cover glass throughout the experiment. 50 μM of probe suspended in 0.2 mL PBS was injected with a 27 gauge needle into the portal vein.

Multiphoton imaging was performed using the Lavision Biotec Nikon multiphoton microscopy (LaVision BioTec, Bielefeld, Germany) and DermaInspect system (Jen-Lab GmbH, Jena, Germany) equipped with an ultrashort (85 fs pulse width) pulsed mode-locked 80-MHz titanium sapphire laser (MaiTai, Spectra Physics, Mount View, CA, USA). The excitation wavelength was set to 740 nm for organ autofluorescence and 850 nm for probe signals, with emission signal ranges of 350 to 450 nm and 515 to 620 nm established respectively through the use of BG39 bandpass filters (BG39, Schott glass color filter, Schott MG, Mainz, Germany). Images were recorded with water-immersion × 10 or oil-immersion × 40 objectives (Carl Zeiss, Oberkochen, Germany). The laser power was set to 20 or 15 mW for × 10 or × 40 magnification imaging, and the acquisition time for obtaining the images was 7.4 seconds per frame. For FLIM, a time-correlated single-photon counting (TCSPC) SPC-830 detector (Becker & Hickl, Berlin, Germany) was incorporated into the DermaInspect system. The TCSPC module constructs a photon distribution across the x and y coordinates of the scan area. Fluorescence emission was spectrally resolved between linearly arranged photon counters through the use of dichroic filters in the beam path. The emission light was collected spectrally in a channel from 515 to 620 nm at 850-nm excitation. Imaging depth of 50 to 100 μm below the fibrous capsule of Glisson were chosen and kept constant throughout this study. Twenty-four images from twelve non-overlapping fields were collected per mouse (*n* = 5) without the use of randomization and blinding.

FLIM images were analysed using SPCImage software 4.9.7 (Becker & Hickl, Berlin, Germany). The fluorescence intensity decay curve of each pixel was fitted to a bi-exponential decay model: *F(t*) = α_1_*e*^−t/τ1^ + α_2_*e*^−t/τ2^ + *C*. Two lifetimes, τ_1_ and τ_2_ represent the fast and slow decay lifetimes; α_1_ and α_2_ are the corresponding relative amplitude coefficients, where α_1_ + α_2_ = 1. *C* is a constant related to the level of background light present and the contribution from all preceding excitation pulses (the ‘*offset*’-signals)^24^,^48^. The values of τ_1_, τ_2_, α_1_, α_2_ and *C* were obtained from the fitting. When excited by laser pulses with an 80 MHz repetition rate, the slow decay fluorescence of the long-lifetime fluorescent probes for hundreds of excitation pulses accumulates and forms a quasi-continuous background. This background is significantly larger than the background caused by possible incomplete decay of endogenous fluorescence. The ‘*offset*’-signals of reacted probes and the cellular autofluorescence (‘*offset*’ parameter within BH SPCImage determines the baseline of the fluorescence decay curve) were adjusted under the chosen measurement conditions that were kept constant throughout this study. Since the fluorescence decay of reacted probes is slow compared to the measurement time window which is defined by the repetition rate of the laser system, the “Incomplete Model” in SPCImage software was used for calculation according to the software handbook.

### Single-cell analysis of images

The cellular-level image analysis was done using ImageJ 1.44p (National Institutes of Health, USA) and Cell Profiler 2.1.0 in Matlab R2015a (The MathWorks Inc.). Grayscale FLIM images were imported and cell pixels were smoothed. The resulting round objects between 30 and 70 pixels in diameter were segmented and saved as the cell within the image. An Otsu Global threshold was used to improve propagation and prevent propagation into background pixels. Then corresponding fluorescence intensity images were imported. Subpopulation analysis was performed by generating histograms of all cell values from fluorescence intensity images of positive cells identified by FLIM images. Each histogram was fitted to 1 and 2 component Gaussian curves. The lowest Akaike information criterion (AIC) signified the best fitting probability density function for the histogram. Probability density functions were normalized to have an area under the curve equal to 1. The OSI was calculated as the ratio of the mean intensity of cellular ROS to the mean intensity of cellular GSH.

### Tissue collection and plasma biochemical measurements

Mouse blood (0.2 mL) was collected in lithium heparin tubes from the inferior vena cava using a 30 gauge needle. At the end of the experiment, the liver, kidney and spleen were excised, and portions were immersed into 10% buffered formalin for histological assessment. Plasma concentration of alanine aminotransferase (ALT) was measured using a Hitachi 747 analyzer (Hitachi Ltd., Tokyo, Japan).

### Histology

Organ specimens were fixed in 4% buffered formalin and embedded in paraffin. Sections were obtained for Hematoxylin & Eosin (H&E) stain to evaluate histopathologic features. The OlyVIA software 2.6 (Olympus, Münster, Germany) was used to visualise and scan the slides. Bromobimane was used for labelling GSH in liver sections on slides according to the manufacturer instructions.

### Statistical tests

Student’s t test with a Bonferroni correction was used to compare the data between groups. All the statistical analysis was done using GraphPad Prism v 6.04 (GraphPad Software Inc., La Jolla, California). Results were considered statistically significant with a *p*-value < 0.05.

## Additional Information

**How to cite this article**: Wang, H. *et al*. Two-photon dual imaging platform for *in vivo* monitoring cellular oxidative stress in liver injury. *Sci. Rep.* **7**, 45374; doi: 10.1038/srep45374 (2017).

**Publisher's note:** Springer Nature remains neutral with regard to jurisdictional claims in published maps and institutional affiliations.

## Supplementary Material

Supplementary Information
