# Supplementary material for: Two-photon dual imaging platform for in vivo monitoring cellular oxidative stress in liver injury
Source: Sci Rep. 2017 Mar 28;7:45374. doi: 10.1038/srep45374 (PMC5368978; doi:10.1038/srep45374)
Supplement: Supplementary Information [file srep45374-s1.pdf]

Two-photon dual imaging platform for *in vivo* monitoring cellular oxidative stress in liver injury

Haolu Wang, Run Zhang, Kim R. Bridle, Aparna Jayachandran, James A. Thomas, Wenzhu Zhang, Jingli Yuan, Zhi Ping Xu, Darrell H. G. Crawford, Xiaowen Liang, Xin Liu, Michael S. Roberts

Supplementary Figures

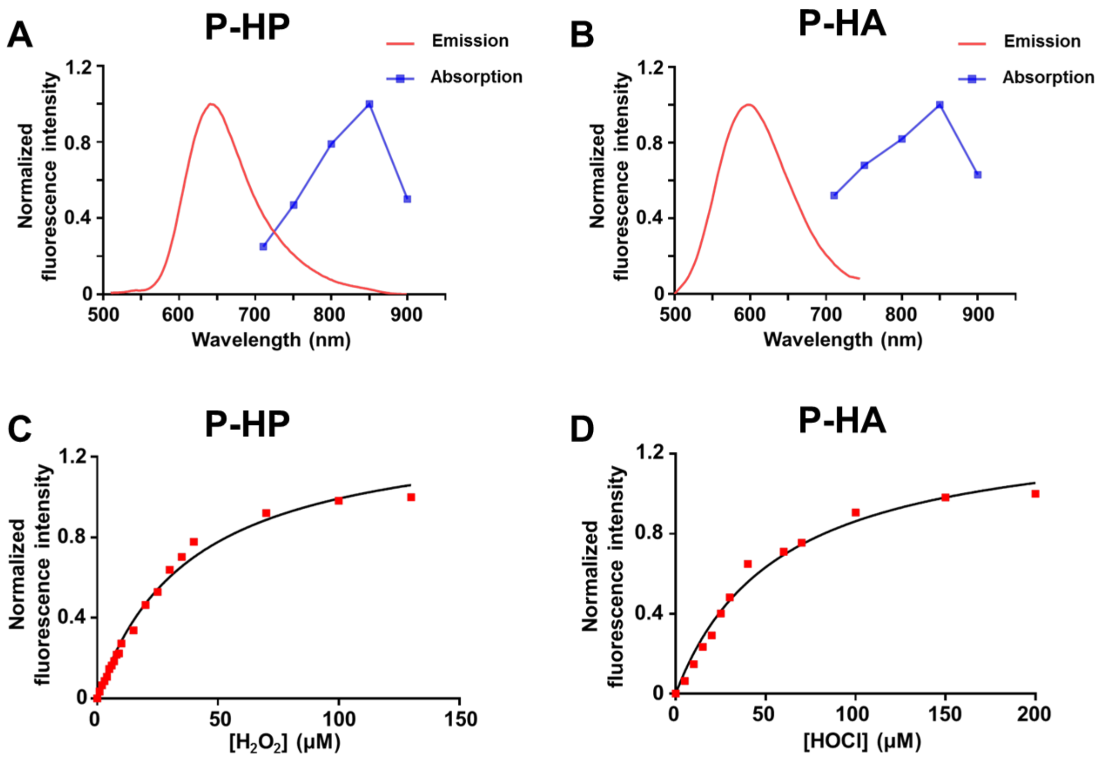

Fig. S1. Spectral characterization of P-HP and P-HA *in vitro*. **(A, B)** Two-photon absorption and emission spectra of P-HP and P-HA reacted with 50 μM of H_2O_2 and 40 μM of HOCl in PBS buffer, respectively. **(C, D)** Fluorescence response of P-HP and P-HA (10 μM) to varying concentrations of H_2O_2 and HOCl, respectively. Values are the mean for $n = 5$ replicates.

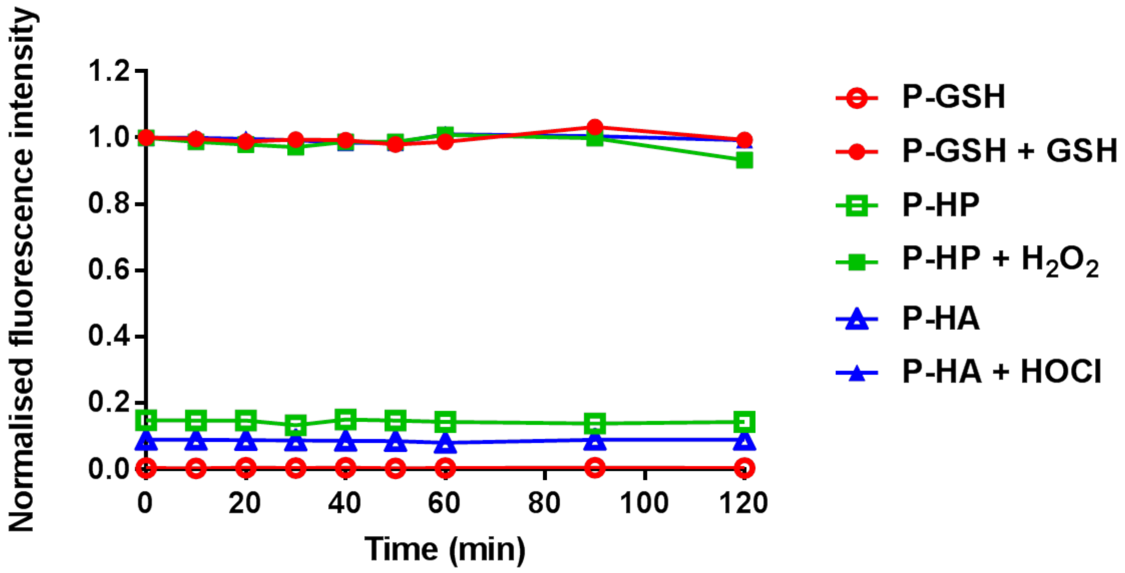

Fig. S2. Signal stability of P-GSH, P-HP and P-HA *in vitro*. Probes were incubated in PBS under the irradiation with a 30 W deuterium lamp at room temperature, and fluorescence was assessed over time. There was no significant change in the fluorescence intensity during the 4 hours of irradiation. Values are the mean for $n = 5$ replicates.

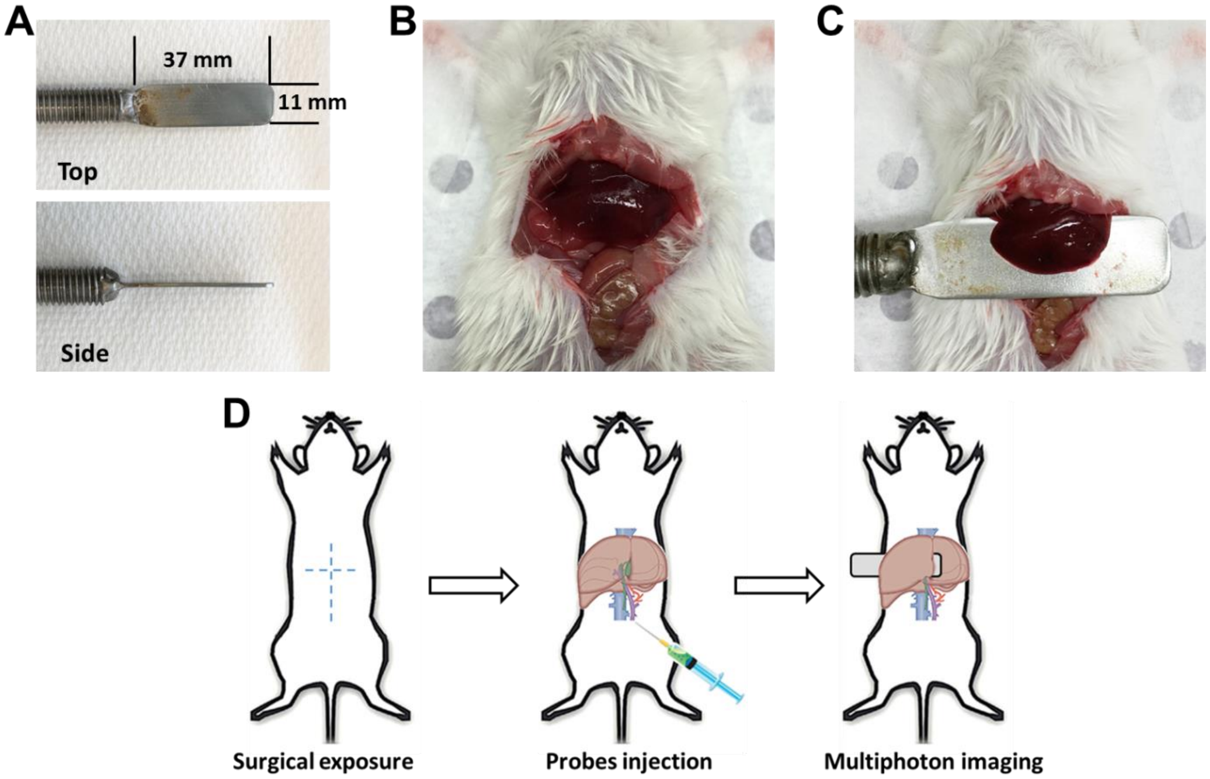

Fig. S3. *In vivo* imaging of cellular ROS and GSH in mouse liver. **(a)** Top and side views of the metal plate for imaging the liver using multiphoton microscopy. **(b)** A midline laparotomy is performed and the liver is exposed for imaging. **(c)** For *in vivo* imaging, the left lobe of the liver is placed on the metal plate, which attaches to an adjustable stand that could be elevated or lowered as required. **(d)** Schema for dual-mode imaging of cellular oxidative stress using metal complex-based probes.

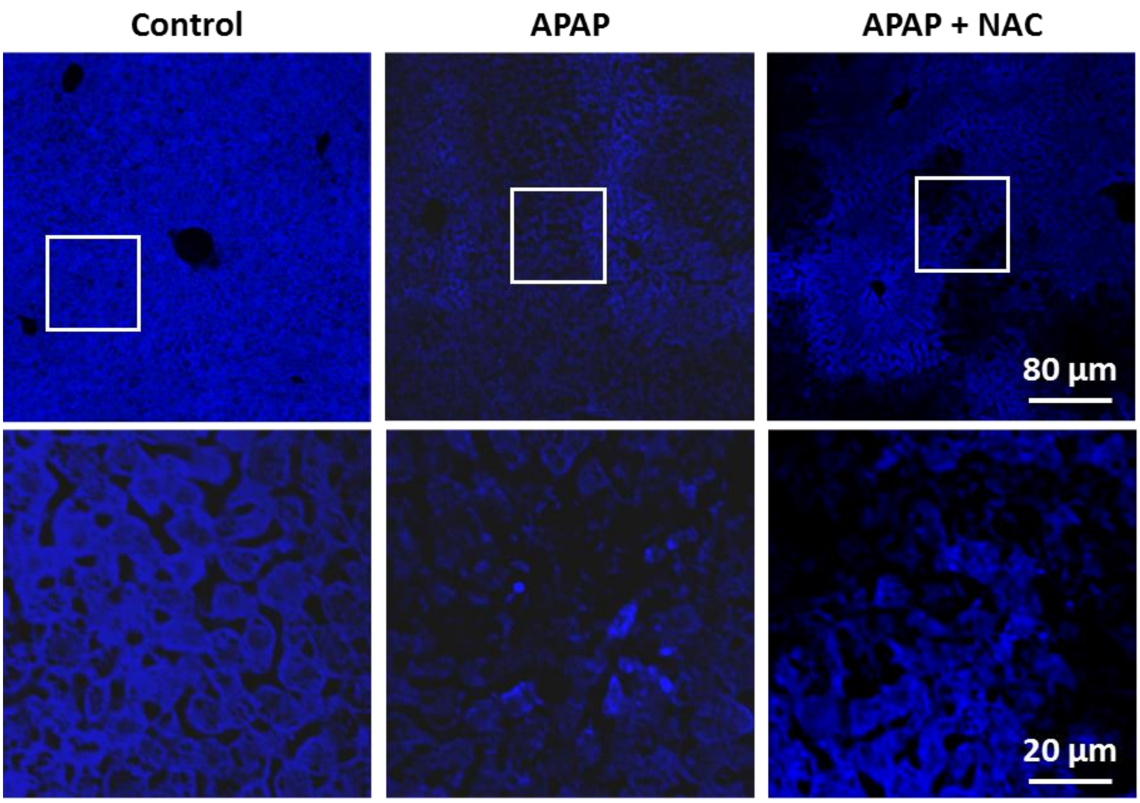

Fig. S4. Representative fluorescence intensity images of liver sections stained by Bromobimane (top), with corresponding image enlargements (bottom). The change of GSH intensity was observed after NAC treatment, correlating well with that *in vivo* detected using our metal complex-based probes.

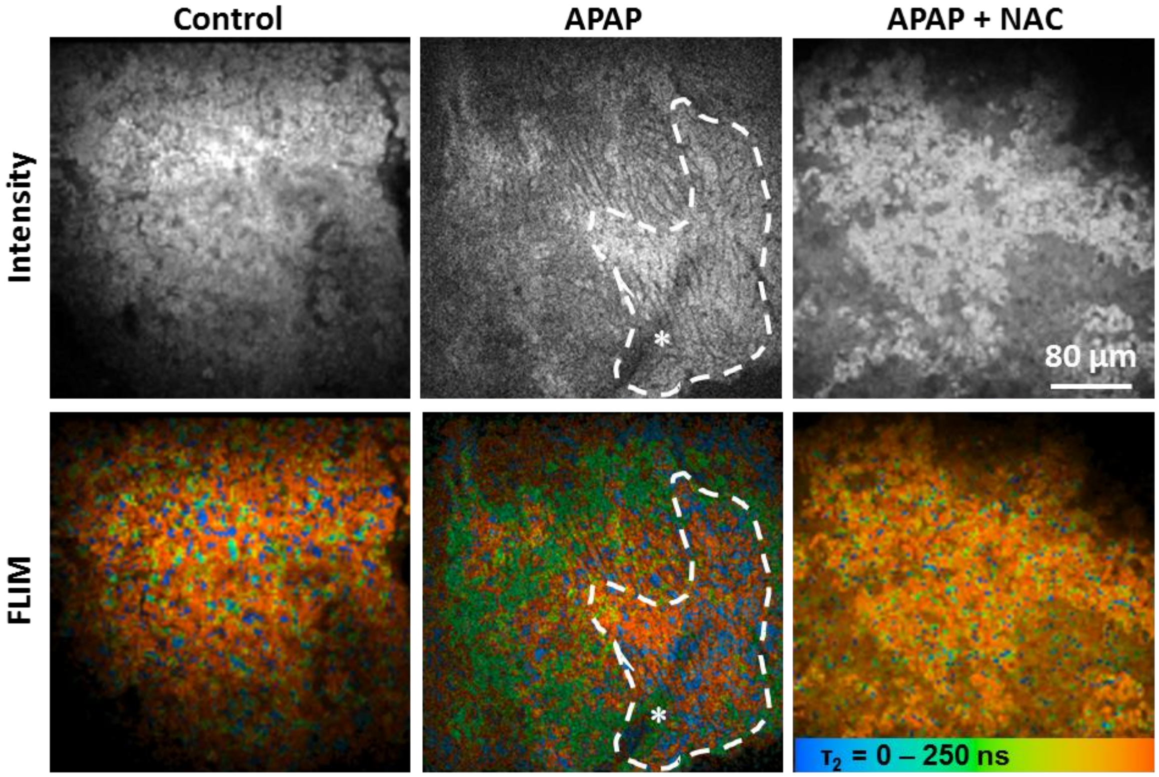

Fig. S5. Dual-mode quantitative imaging of the change of GSH in hepatocytes responses to NAC treatment against APAP induced liver injury at low magnification (10×). Hepatocytes around the portal vein (circled area) are less sensitive to APAP-induced GSH decrease. All images were recorded at $\lambda_{Exc}/\lambda_{Em}$: 850/515 to 620 nm. Asterisks indicate portal vein. Scale bar: 20 µm.

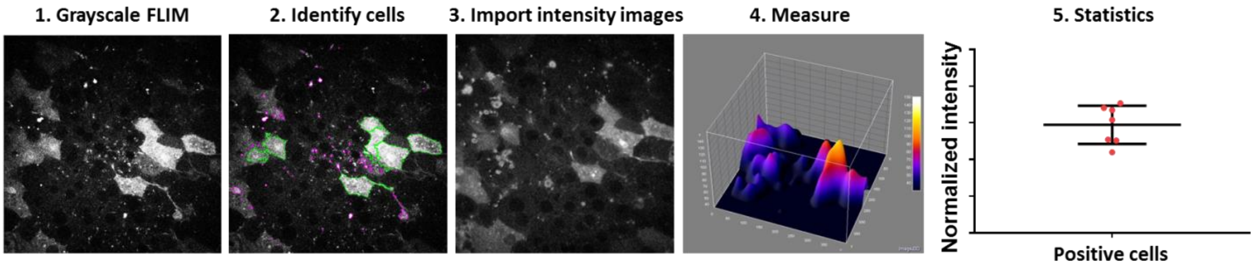

Fig. S6. Single-cell analysis of high-resolution fluorescence intensity images.

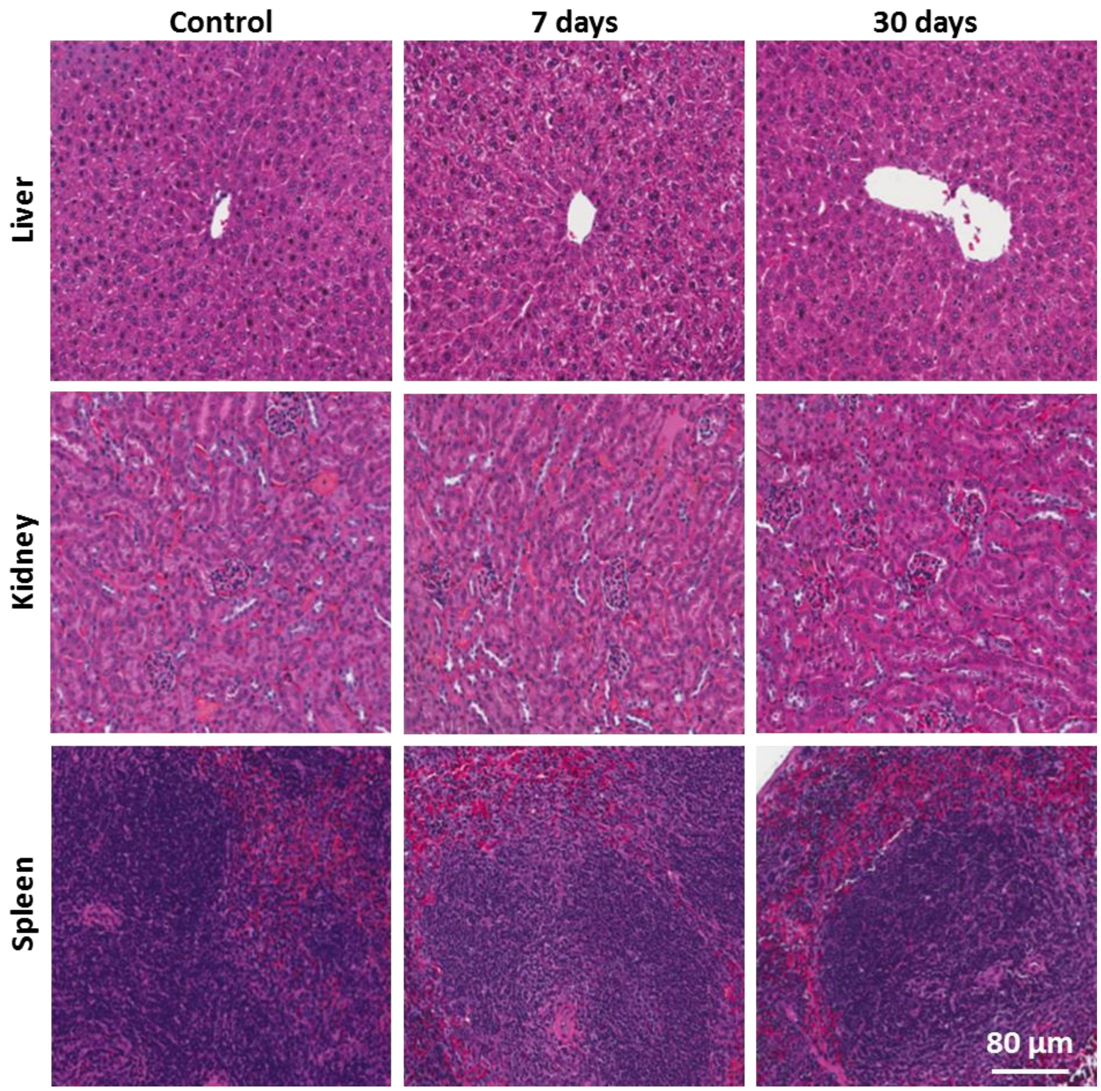

Fig. S7. Representative histology (H&E staining) of major organs of mice after injection of 50 μM of probes. No obvious necrosis and abnormity were observed in the sections of liver, kidney and spleen by histological examination. Scale bar: 80 μm.

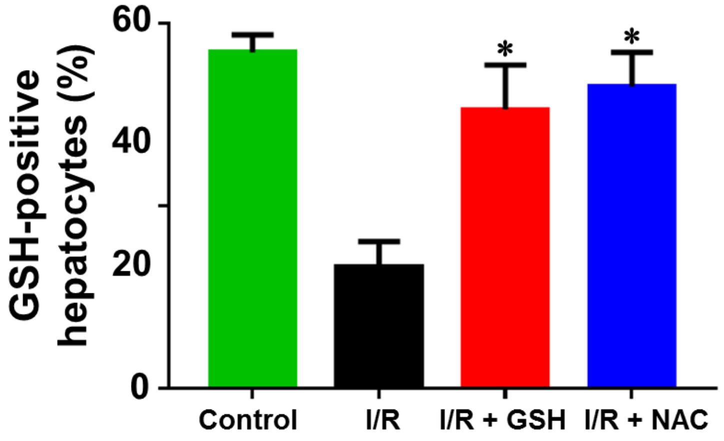

Fig. S8. The percentages of GSH-positive hepatocytes in all groups. Values are the mean ± s.d. for $n = 5$ mice; * $p < 0.05$, compared with untreated groups.

Supplementary Tables

Table. S1. Optical characteristics of P-GSH, P-HP and P-HA

Probe	Two-photon absorption peak (nm)	Emission peak (nm)	Fluorescence lifetime (ns)*
P-GSH	850	612	225
P-HP	850	645	146
P-HA	850	600	90.5

*: After reacted with GSH, H_2O_2 or HOCl

Table. S2. The percentages of GSH-positive hepatocytes determined by P-GSH and bromobimane

Group	P-GSH (%)	Bromobimane (%)
Control	85.4 ± 12.7	82.9 ± 9.8
APAP	19.3 ± 4.2	13.5 ± 3.1
APAP + NAC	77.9 ± 11.4	74.0 ± 12.3
